# Current Understanding of Immunological Skin Diseases: Atopic Dermatitis, Generalized Anhidrosis, and Drug Hypersensitivity

**DOI:** 10.3390/ijms23147563

**Published:** 2022-07-08

**Authors:** Hideo Hashizume

**Affiliations:** Department of Dermatology, Iwata City Hospital, 512-3 Ohkubo Iwata, Shizuka 438-8550, Japan; hihashiz0001@mac.com; Tel.: +81-538-38-5000; Fax: +81-538-38-5050

**Keywords:** atopic dermatitis, generalized anhidrosis, drug-induced hypersensitivity syndrome

Recent dermatological research has progressed, particularly novel technologies and analytical methodologies, providing great advances in the exploration of previously poorly understood interactions between the skin—the outermost surface of humans—and the external environment. The skin is an essential physiological and immunological barrier that maintains homeostasis in humans [1]. Because the skin is in constant contact with the external environment, it is always exposed to various stimuli, which may disrupt its barrier functions leading to continuous repair, and in some cases, it never recuperates, causing persistent inflammation, which may involve the deeper tissues leading to hypersensitivity. Here, we present six excellent articles in this Special Issue titled “Skin Allergy and Immunology”.

During our evolution from aquatic animals to land-dwelling creatures, humans have evolved skin, our outermost surface, which retains water in the body. Water is essential for survival because it is critical for the functions of biomolecules. The cornification of keratinocytes, alternatively armored with dead cells, prevents water loss from viable cells. Indeed, a fetus at 14 weeks gestation starts cornification to prepare for birth from the womb into the unexperienced external world [2].

Gallegos-Alcala et al. [3] summarized the physiology and pathology of keratinocytes, which are the main components of the epidermal barrier. The physiological functions of keratinocytes can be classified into three phases. In the proliferation phase, basal cells divide, maintaining adhesion; in the maturation phase, these cells begin migration through the strata of the epidermis with alterations in keratin expression from K5/K14 to K1/K10; in the cornification phases, these cells release lipids, proteins, and enzymes to prepare for desquamation and form a cornified cell envelope. In the last cornification phase, these cells die and form a physiological barrier with filaggrin in granular cells, which is genetically involved in the pathogenesis of atopic dermatitis (AD). Filaggrin is catabolized into hygroscopic amino acids that function as natural moisturizing factors and degradation products that protect against ultraviolet B damage.

Although cornification is a barrier to water transpiration, the complete shielding of water increases the body’s temperature. Therefore, the perspiration system in the skin functions as a radiator to maintain an optimal body temperature for biomolecule functions. Kageyama et al. [4] reported on acquired idiopathic generalized anhidrosis, a rare but serious disorder, characterized by the sudden onset of systemic anhidrosis/hypohidrosis without direct causative diseases, which is frequently accompanied by cholinergic urticaria. They reviewed the current knowledge of its pathogenetic mechanisms, including the association between perspiration and mast cell activation. They also indicated that this disease is frequently associated with the emergence of several autoimmune disorders, suggesting an autoimmune etiology.

The skin should protect against the entry of invading pathognomonic microorganisms. Therefore, skin also has an important role as an immunological barrier as reviewed by Gallegos-Alcala et al. [3]. Furthermore, the skin contains various immune-competent cells including T- and B-lymphocytes, γδT-cells, innate lymphoid cells, macrophages, Langerhans cells, dermal and plasmacytoid dendritic cells, and mast cells, with the transient recruitment of special cell subsets including neutrophils, eosinophils, and basophils in cases of local inflammation. Interestingly, keratinocytes also have some immunological functions that augment inflammatory responses by producing biomolecules including cytokines, chemokines, and alarmins. Nerves in the skin are in direct contact with mast cells, which indirectly impact various immune cell types by secreting neuropeptides that modulate inflammation. Gallegos-Alcala et al. explained the close relationship between the skin barrier and immune responses in the pathogenesis of AD. Even in AD patients without filaggrin gene mutations, a Th2/Th22 signature abrogates the expression of filaggrin in the epidermis. Thus, a defective barrier function is highly associated with Th2-shifted immune responses and vice versa.

Allergy is a hypersensitivity reaction to substances that are harmless to most people. Skin is frequently associated with allergic reactions that are often related to the concentration of various immunocompetent cells eliminating internal pathogenic substances to the external side of the skin. Drug hypersensitivity is the most frequent iatrogenic disorder, some of which are life threatening. Miyagawa et al. [5] discuss the recent advances in drug-induced hypersensitivity syndrome/drug reactions with eosinophilia and systemic symptoms (DIHS/DRESS), a severe cutaneous adverse reaction characterized by high fever, erythroderma, facial edema, lymphadenopathy, eosinophilia, and multiple organ damage. Of note, latent infections of herpes viruses including human herpesvirus-6 (HHV-6) and cytomegalovirus are frequently reactivated during the disease course. Miyagawa et al. also report on recent studies investigating the mechanisms related to the pathogenesis of DIHS/DRESS, with a focus on HHV-6 reactivation.

Additionally, this volume contains three original articles: two on AD and one on DIHS/DRESS. Park et al. [6] investigated *Cera Flava*, a natural extract from beehives, for the treatment of AD. They demonstrated that the topical application of *Cera Flava* alleviated atopic inflammation in NC/Nga mice, an AD mouse model, presumably via the TLR2/ERK pathway. Because this agent has been widely and safely used for a long time in the dermatological field, it might be a potential option for AD treatments in the future. Lee et al. [7] report a novel alternative mechanism involved in AD pathogenesis. The SWItch3-related gene product, an SW1/Sucrose Non-Fermenting chromatin remodeling subunit, alternatively termed the BAF complex, is an epigenetic regulation factor that remodels chromatin. This has been well investigated in oncogenesis; however, there have been few studies of its role in other disorders. This report provides a novel perspective on AD pathology. Toniato et al. [8] conducted a retrospective study to investigate the comorbidities and therapies in 25 DRESS cases at their institute. They demonstrated that the number of daily medications and elder age (≥68 years) was associated with disease severity and found several previously unknown predisposing elements to this disease.

In conclusion, these articles in the present Special Issue demonstrate the critical importance of skin, which is only 2 mm thick. These reviews highlight the exquisite physiological systems present in the skin as well as pathological mechanisms related to skin allergic diseases, which have gained increasing attention recently. With the assistance of evolving modern technology and medicine, we now understand the pathogenetic mechanisms of immunological skin diseases better, providing novel strategies for their treatment.
